# Pneumoparotid and Pneumoparotitis: A Literary Review

**DOI:** 10.3390/ijerph17113936

**Published:** 2020-06-02

**Authors:** Francesco Gazia, Francesco Freni, Cosimo Galletti, Bruno Galletti, Rocco Bruno, Cosimo Galletti, Alessandro Meduri, Francesco Galletti

**Affiliations:** 1 Department of Adult and Development Age Human Pathology “Gaetano Barresi”, Unit of Otorhinolaryngology, University of Messina, 98125 Messina, Italy; franco.freni@tiscali.it (F.F.); cosimogalletti92@gmail.com (C.G.); bgalletti@unime.it (B.G.); brunor@unime.it (R.B.); fgalletti@unime.it (F.G.); 2 Comprehensive Dentistry Department, Faculty of Dentistry, Universitat de Barcelona, L’Hospitalet de Llobregat (Barcelona), 08907 Catalonia, Spain; cosimo88a@gmail.com; 3 Department of Scienze Biomediche, Odontoiatriche e Delle Immagini Morfologiche e Funzionali, Unit of Ophthalmology, University of Messina, 98125 Messina, Italy; ameduri@unime.it

**Keywords:** pneumoparotid, pneumoparotitis, parotitis, Stensen’s duct, head and neck

## Abstract

Pneumoparotid is a rare condition of parotid swelling. The presence of the air in gland parenchyma is caused by an incompetent Stensen’s duct with high pressure may cause the acini’s rupture. We reviewed 49 manuscripts, from 1987 to today, that enrolled a total of 54 patients with pneumoparotid. Our review evaluated the following evaluation parameters: gender, age, etiology, clinical presentation, treatment, days of resolution after diagnosis, relapse and complications. The most frequent etiology is self-induction by swelling the cheeks (53.7%). This cause mainly involves children (74%), for conflicts with parents, excuses for not going to school, nervous tics or adults (16%) with psychiatric disorders. Iatrogenic causes are also frequent (16.6%), for dental treatments (55.5%) or use of continuous positive airway pressure (CPAP) (33.4%). Medical therapy is the most practiced (53.7%), in most cases it is combined with behavioral therapy (25.9%) or psychotherapy (25.9%). Surgery is rarely used (9.2%) as a definitive solution through parotidectomy (50%) or ligation of the duct (50%). The most common complication is subcutaneous emphysema (24.1%), sometimes associated with pneumomediastinum (5.5%). Careful treatment and management are necessary to ensure the resolution of the pathology and counteract the onset of complications.

## 1. Introduction

**Figure 1:**
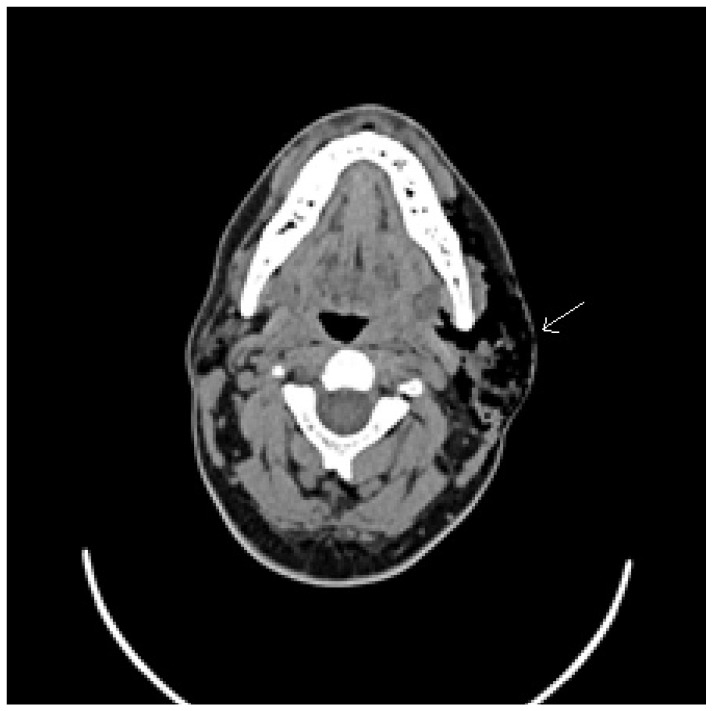
Axial projection computed tomography (CT) image of a left pneumoparotid case, with the arrow indicating the presence of air in the parotid lodge.

Pneumoparotid is a rare cause of parotid enlargement due the presence of air within the parotid gland. The pneumoparotid term, first described in 1865 by Hyrtl, defines the presence of air within parotid system: gland and Stensen’s duct [1]. The condition was recognized also in 1915 when a strange epidemic of mumps occurred in the French Foreign Legion in North Africa. The soldiers were deliberately self-inducing the condition by blowing into a small bottle to avoid duty [2]. Conditions that increase intraoral pressure like Valsalva’s maneuver or incompetent Stensen’s duct are predisposing factor to pneumoparotid. Pneumoparotitis is a complication of pneumoparotid that proceeds towards an inflammatory state or infection process. In general, local pain in the parotid area and swelling are the most common symptoms. We have noticed how often in the literature pneumoparotid and pneumoparotitis are used interchangeably. In reality, the latter is a complication of the former. In our review, we clarified the real percentage of this complication. Subcutaneous emphysema has been described as a complication of this condition and occurs from an extension of the air leak from the affected parotid acini to the surrounding cervicofacial subcutaneous tissues [3]. Literature shows cases of pneumoparotid in adolescents and adults with psychosocial issues. A correct anamnesis and imaging studies like ultrasound, sialendoscopy and head–neck computed tomography (CT) are essential to perform a correct diagnosis (Figure 1). Treatment generally includes supportive medical management, reserving surgical therapy in case of severe cases [4].

The main problem of pneumoparotid is that it is the clinical condition not well-described in the literature—only clinical reports are published, without any observational study with large numbers of patients, no studies comparing the various treatments or how to prevent complications.

The purpose of our review is to collect all the data present in the literature and make a general analysis on the epidemiology, etiology, treatment and management of this rare disease.

Furthermore, in the literature there are only case reports, we wanted to write the first review to clarify all the salient points of this clinical condition and to provide the scientific community with the correct indications to diagnose and quickly treat pneumoparotid, avoiding complications.

## 2. Materials and Methods

**Figure 2:**
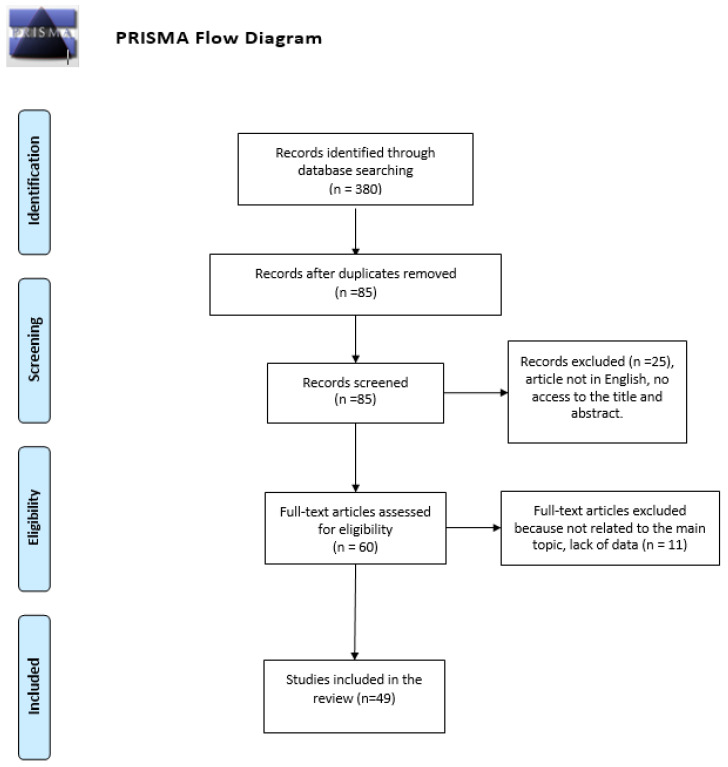
Review preferred reporting items for systematic review (PRISMA) flow diagram.

We have analyzed the case reports or case series in English, full-text access (open access or payment) that have pneumoparotid treatment and management as their main topic. All articles were found on PubMed, Scopus and Web of Science using the keywords “pneumoparotid”, “pneumoparotidis”, “pneumoparotis” and “parotid emphysema” in four different searches. The data of this systematic investigation observed the preferred reporting items for systematic review (PRISMA) accordingly with the statement (Figure 2). We only considered the cases of symptomatic patients, excluding patients with occasional findings (for example after the puffed-cheek maneuver for the CT study of the oral cavity). We reviewed 49 manuscripts, from 1987 to today, that enrolled a total of 54 patients with pneumoparotid. Our review evaluated the following evaluation parameters: gender, age, etiology, clinical presentation, treatment, days of resolution after diagnosis, relapse and complications (Table 1).

## 3. Results

We analyzed a total of 54 patients, 39 males and 15 females. The mean age was 22.3 years, but we can consider a group of patients in scholar age (31 patients, 11.9 years mean age) and a group of adults (23 patients, 40.8 years mean age). The clinical presentation is characterized by swelling of the parotid region sometimes extended to others districts, which can be bilateral (48.1%) or unilateral (51.9%). The most frequent etiology is self-induction by swelling the cheeks (53.7%). This cause mainly involves children (84%), for conflicts with parents, excuses for not going to school, nervous tics or adults (16%) with psychiatric disorders. The cases of idiopathic pathology are 24.1%. Iatrogenic causes are also frequent (16.6%), for dental factors (55.5%), use of CPAP (33.4%) or during spirometry (11.1%). Persistent coughing attacks can also be a cause in subjects with chronic bronchitis (5.5%).

Regarding the treatment, medical therapy is the most practiced (53.7%), with the use of antibiotics and steroidal anti-inflammatories or not. In most cases, medical therapy is combined with other treatments. Behavioral therapy is used to remove bad habits that can lead to this pathology (25.9%), with zeroing the recurrence rate if the subject is collaborative. If the subject has mental disorders, supportive psychotherapy is often required (25.9%), with a prevalence in children (95%). When the pathology does not resolve or tends to be recidive, more invasive approaches are used, such as needle aspiration (3.7%). Surgery is rarely used (11.1%) as a definitive solution through parotidectomy (50%) or ligation of the duct (50%). Corticosteroid infiltration sialoendoscopy was used in 2 cases without success. There were also 3 cases (5.5%) that did not require any treatment for resolution. Regarding our analysis, the pathology resolves in 4.5 days with the appropriate treatment, due to the low number of cases further investigation occurred. The disease relapsed in 23 subjects, but in 3/51 cases no data concerning the recurrence rate was found. From the data we analyzed, the recurrence rate is 42.6%, mainly affecting psychiatric subjects (60%). The most common complication is subcutaneous emphysema (24.1%), sometimes associated with pneumomediastinum (5.5%). Parotitis associated with pneumoparotide, which is called pneumoparotitis, has only been described in 14.8% of cases, underlining an improper use of this term. Abscess of the parotid lodge occurred only once (1.8%) (Table 2).

## 4. Discussion

Pneumoparotid is a very rare condition of parotid gland, often complicating with a subcutaneous emphysema, causing swelling of the parotid lodge. This pathology usually occurred due no physiological stagnation of air in parotid parenchyma. Pneumoparotid is usually associated with a retrograde insufflation of air and saliva via Stensen’s duct into the secondary ducts and glandular acini [40]. Hypotonia of the buccinator muscle, hypertrophy of the masseter muscle or temporary obstruction of the Stensen’s duct by mucous are described as possible risk factors [1].

The opening of the Stensen’s duct lies near to the second upper molar tooth bilaterally. The normal anatomy of duct preventing the reflux of air and saliva into the parotid gland are three fold:

1. The diameter of the duct orifice is smaller than that of the duct itself;
2. The duct opening is covered by redundant mucosal layer, covering the duct orifice when there is increased intraoral pressure;
3. The Stensen’s duct is compressed in its lateral course along the masseter muscle and its passage through the buccinator muscle with an increase in oral pressure.

In our experience, we report a case of a 45-year-old man with numerous episodes of painful, mono lateral left facial swelling. Clinical examination reported left-sided painful and parotid swelling with crepitus. Head–neck CT examination reported very important presence of subcutaneous emphysema that affected caudo-cranial left soft tissues from temporal region to the upper thoracic outlet, severe ectasia of Stensen’s duct, ducts of salivary glands and left parotid (Figure 1). Aware of the patient’s psychiatric conditions, psychiatric counseling is demanding. The colleagues reported that the patient suffered form of a minor cognitive disability with a tendency to somatization, underlying an important state of anxious and insomnia, prescribing a psychiatric therapy with venlafaxine, quetiapine and alprazolam. The patient is treated with antibiotic therapy and support measures with resolution of subcutaneous emphysema and general health condition. Our experience is in agreement with the case studies, management and treatment of the pathology described in the literature.

Medical literature showed a frequent association with glass blowing, playing wind instruments, exercising and self-induced behaviors often linked to psychiatric disorders. Normal intraoral pressure is 2 to 3 mm Hg, in glassblowing and trumpet playing this pressure may increase until 150 mmHg facilitating the disease’s development. Furthermore, iatrogenic pneumoparotid is described like complication of spirometry, odontoiatric procedures, fine needle aspiration of the parotid gland and positive pressure ventilation used preoperatively or in the intensive care setting [11,16,18,27,42,50,51,52]. Long-term use of oronasal continuous positive airway pressure is a potential cause of pneumoparotid [41,44]. Viral and bacterial infections, autoimmune diseases like sarcoidosis, Sjögren syndrome and Wegner’s vasculitis, diabetes, Cushing disease, hypothyroidism, liver disease are described like possible causes of pneumoparotid or pneumoparotidis [2].

Repeated episodes of pneumoparotid may cause to chronic inflammation, infection or sialectasis.

The pathophysiologic condition of pneumoparotid has also been demonstrated by using a “puffed-cheek’’ technique [53], usually performed a CT examination after sialography, which mark filling defects, air in the parotid ductal system and sialoliths. Next, massaging the both patient’s parotid glands, CT scan is performed highlighted a reduced amount of air and absence of contrast. Repeated maneuvers of autoinflation with high pressure may cause the acini’s rupture. As we know the parotid’s capsule is incomplete in the superiomedially part at the posterior border of mandible bone, airflow could reach the parapharyngeal and retropharyngeal space [2], provoking emphysema.

Enlargement of the parotid gland may be due to mumps, bacterial sialadenitis, obstructive sialadenitis, autoimmune disease like Sjogren syndrome. There are also rarer causes that can lead to swelling of the parotid, for example tuberculosis, sarcoidosis, cat-scratch disease or trauma. Pneumoparotid refers to the pathologic state of air within the parotid gland with or without inflammation. The clinical history of the patient (glass blowing, playing wind instruments, self- induced behaviors often linked to psychiatric disorders) and radiodiagnostics play a crucial role in the differential diagnosis. Pneumoparotid should be suspected with painless or minimally painful parotid swelling in the absence of fever. In the acute phase, plain radiographs may show air within the ductal system, sometimes with extravasation into the parenchyma and surround soft tissues. Computed tomography demonstrates air contrast with great sensitivity. Ductal dilation is a common finding on both sialography and computed tomography.

Imaging techniques are essential to perform a correct diagnosis. In reviewing the medical literature, radiologic studies that are indicated as good practice are ultrasonography, sialography, radionuclide sialography, sialendoscopy, salivary gland isotope scanning, CT and nuclear magnetic resonance (NMR) [54]. The use of ultrasound is stronglyrecommended in the diagnosis of superficial swelling in the head–neck area in general, and for salivary gland diseases in particular. It marks multiple hyperechoic areas corresponding to air in the glandular parenchyma, ducts and soft tissue. It is easy, reliable, non-invasive, cost-efficient and provides real-time conservative dynamic imaging. Scialography is useful for establishing the presence of stones, although less sensitive. [2,4].

In recent last years, sialendoscopy has become a good routine technique and minimally invasive diagnostic procedure of the parotid gland. The main goal is the evaluation and management of the salivary ductal system [40]. Currently, CT is the gold-standard technique because it defines anatomy and it is not invasive.Describing air-filled dilatation of Stensen’s duct, glandular acini air dilatation, collections, free air intraparenchymal and a good imaging of duct glandular system, also helps in diagnoses of extension of air-accumulation in the nearest areas of the head–neck district [2]. Puffed-cheek CT is a good technique that demonstrated a subtle, but definite increase in intraductual and intraglandular parotid air when is compared to the simple CT [53].

Clinical treatment is the first step in approaching pneumoparotid. Acute management includes a short line of antibiotics, oral or intravenous, with the addition of steroids if the swelling is severe.

Antibiotics are used to protect the host from secondary infections; analgesia is also considered to improve general health state of patient. A parallel line of treatment includes massage of the gland, hydration, mouthwashes, sialogogues and warm compresses. In self-induced pneumoparotid cases, psychotherapy is necessary to correct the underlying adaptative psychiatric disorder. In severe cases or recurrences—sometimes associated with infection or pneumomediastinum—surgery is required: glandular resection, ductoplastic and/or Stensen’s duct ligation, partial parotidectomy with duct’s ligation. Parotid duct ligation is considered as a gold-standard for recurrent or chronic severe parotid infection. Parotidectomy is required in rare cases, usually when the patient is noncompliant, in failure of treatment or chronic infection, is the end point line of treatment [2,9,20,29,30,55].

Parotidectomy is an invasive surgery procedure that can induced complications that patients and professionals have to considered: partial or complete facial nerve lesion, Frey’s syndrome [56,57], salivary fistula, auricularis magnus nerve lesion and keloid cicatrization of surgery incision. To avoid the recurrences of pneumoparotid a counseling to explain that are it is essential to stop activities that increase intraoral pressure is already fundamental.

The limit of our review is represented by the fact that all the selected articles are case reports or case series. There are no observational, retrospective or prospective studies in the literature. This review may be a starting point for clinical studies with a larger number of patients. Given the lack of comparative studies between the various therapeutic treatments or on the prevention of complications, further studies are needed for the definition of guidelines or gold-standard.

## 5. Conclusions

Pneumoparotid is not a real pathology, but a non-physiological clinical condition characterized by the presence of air in the Stensen’s duct and throughout the gland—and can be complicated. Pneumoparotid affects two target populations, children and adults. Thanks to this review, we have clarified some important aspects concerning the etiopathogenesis and pathophysiology of pneumoparotid. We pointed out that the most frequent cause is self-induction, caused most often by people with psychiatric disorders. Regarding the treatment, there is no gold-standard, but each patient must be treated according to his/her clinical condition, speeding up the diagnostic process through a CT examination. In case of complications such as pneumoparotitis, antibiotic therapy is indispensable. In the complication of subcutaneous emphysema, the clinic, the size and the recurrence rate must always be evaluated to avoid the evolution towards pneumomediastinum. In case of critical dimensions, needle aspiration or surgical treatment is appropriate. In case of recurrence, more aggressive surgical treatment should be considered. Careful treatment and management are necessary to ensure the resolution of the pathology and counteract the onset of complications.

## Figures and Tables

**Table 1:** Manuscripts analyzed.

Manuscript	Sex	Age	Clinical Presentation	Etiology	Treatment	Resolution after Diagnosis	Relapse	Complication
Garber et al., 1987 [5]	M	32	Bilateral	Hay fever (Coughing attack)	Medical	5 days	No	
Markowitz et al., 1987 [6]	F	12	Bilateral	Self-induced	Medical and psychotherapy	1 day	Yes	
David et al., 1988 [7]	F	6	Left	Self-induced	Medical, needle aspiration and psychotherapy		No	Parotitis
Brodie et al., 1988 [8]	M	14	Bilateral	Self-induced	Surgery (transposition of the duct)		No	Subcutaneous emphysema
Telfer et al., 1989 [9]	M	29	Right	Idiopathic	Surgery (treatment of drooling second Brody)		No	/
Mandel et al., 1991 [10]	M	53	Right	Self-Induced	Behavioral	1 day	Yes	/
Piette et al., 1991 [11]	F	34	Right	Iatrogenic (dental care)	Medical	5 days	No	/
Takenoshita et al., 1991 [12]	M	24	Left	Iatrogenic (dental care)	Medical	2 days	No	/
Krief et al., 1992 [13]	M	10	Bilateral	Self-induced	Medical and psychotherapy	/	Yes	Parotitis
Curtin et al., 1992 [14]	M	26	Bilateral	Self-Induced	Behavioral	/	No	/
Ferlito et al., 1992 [15]	M	14	Bilateral	Self-Induced	Medical and psychotherapy	/	Yes	
Brown et al., 1993 [16]	M	30	Left	Iatrogenic (Air-powder prophylaxis units for removing plaque)	Medical	5 days	No	/
Birzgalis et al., 1993 [17]	M	16	Right	Self-Induced	Behavioral	/	No	Subcutaneous emphysema
McDuffie et al., 1993 [18]	M	24	Bilateral	Iatrogenic Orthodontic appliances	Behavioral (correction of orthodontic appliances)	3 days	No	/
Cook et al., 1993 [19]	F	44	Bilateral	Coughing attack	None		No	
Nassimbeni et al., 1995 [20]	M	12	Bilateral	Self-induced	Psychotherapy		No	Subcutaneous emphysema
	M	9	Right	Self-induced	Surgery (parotidectomy)		Yes	Abscess
Goguen et al., 1995 [21]	M	9	Right	Self-induced	Behavioral	1 day	No	
	F	9	Bilateral	Self-Induced	Medical and psychotherapy		Yes	Parotitis
	M	13	Bilateral	Self-Induced	Psychotherapy		Yes	
Ros et al., 1996 [22]	M	3	Left	Self-Induced	None	1 day	No	
Gudlaugsson et al., 1998 [23]	F	16	Bilateral	Self-induced	Medical and psychotherapy		Yes	Subcutaneous emphysema, pneumomediastinum
Alcalde et al., 1998 [24]	M	29	Right	Idiopathic	Needle aspiration, medical and bite		No	
Golz et al., 1999 [25]	M	10	Bilateral	Self-induced	Psychotherapy		No	
Sittel et al., 1999 [26]	F	14	Bilateral	Self-induced	Medical and psychotherapy		Yes	
Kirsch et al., 1999 [27]	M	41	Left	Iatrogenic Spirometry	None	1 day	Yes	
Martín-Granizo et al., 1999 [28]	F	5	Bilateral	Idiopathic	Medical	2 days	Yes	
	F	8	Right	Coughing attack	Medical			
Han et al., 2004 [29]	M	13	Rigt	Self-induced	Medical and surgery (duct ligation)	2 days	No	Subcutaneous emphysema
Apaydin et al., 2004 [30]	M	50	Left	Idiopathic	Surgery (parotidectomy)	After surgery	No	
Grainger et al., 2006 [31]	F	12	Bilateral	Idiopathic	Medical	2 days	Yes	
Balasubramanian et al., 2008 [3]	M	11	Bilateral	Self-induced	Medical and psychotherapy		Yes	Subcutaneous emphysema
Prabhu et al., 2008 [32]	M	12	Bilateral	Self-induced	Medical and psychotherapy		Yes	
Luaces et al., 2008 [33]	M	11	Right	Self-induced	Medical	28 days	Yes	Subcutaneous emphysema
Faure et al., 2009 [34]	M	9	Left	Self-induced	Medical and psychotherapy		Yes	
Kyung et al., 2010 [35]	F	7	Bilateral	Self-induced	Medical and behavioral	3 days	No	Subcutaneous emphysema, pneumomediastinum
Zuchi et al., 2011 [1]	F	50	left	Idiopathic	Medical	14 days	No	Parotitis
Mukundan et al., 2011 [36]	M	13	Left	Self-induced	Medical		No	
van Ardenne et al., 2011 [37]	F	7	Right	Self-induced	Medical and Behavioral	30 days	No	
Ghanem et al., 2012 [38]	M	46	Unilateral	Idiopathic	Sialendoscopy		Yes	Parotitis
Potet et al., 2012 [39]	F	44	Left	Idiopathic	Medical		No	Parotitis
McGreevy et al., 2013 [2]	M	48	Right	Idiopathic	Surgery (parotidectomy)	After surgery		Parotitis (before surgery)
McCormick et al., 2013 [4]	M	7	Bilateral	Idiopathic	Medical		Yes	
Konstantinidis et al., 2014 [40]	M	61	Right	Idiopathic	Sialendoscopy with corticosteroids		Yes	Parotitis
Abdullayev et al., 2014 [41]	M	36	Bilateral	Iatrogenic CPAP	Behavioral (stopping CPAP)	1 day	No	
Cabello et al., 2015 [42]	M	42	Right	Iatrogenic MAD	Behavioral (regulating MAD)		No	
Alnæs et al., 2017 [43]	F	10	Left	Self-induced	Medical and behavioral	1 day	Yes	Subcutaneous emphysema
Goates et al., 2017 [44]	M	53	Left	Iatrogenic CPAP	Behavioral (nasal CPAP)	1 day	No	
	M	54	Right	Iatrogenic CPAP	Behavioral (nasal CPAP)	1 day	No	
Lagunas et al., 2017 [45]	M	13	Bilateral	Self-induced	Medical and behavioral	1 day	Yes	Subcutaneous emphysema
Yamazaki et al., 2017 [46]	M	53	Bilateral	Self-induced	Medical and behavioral		No	
Lee et al., 2018 [47]	M	11	Bilateral	Idiopathic	Medical	4 days		Subcutaneous emphysema, pneumomediastinum
House et al., 2018 [48]	M	34	Bilateral	Self-induced	Medical and psychotherapy		Yes	Subcutaneous emphysema, parotitis
Ambrosino et al., 2019 [49]	M	12	Bilateral	Idiopathic	Medical		Yes	Subcutaneous emphysema

**Table 2:** Summary of results.

Results	M ± SD *n* (%)
**Gender**	
Male	39/54 (72.2%)
Female	15/54 (27.8%)
**Age** (Years)	22.3 ± 17.7
**Clinical Presentation**	
Bilateral	26/54 (48.1%)
Monolateral	28/54 (51.9%)
**Etiology**	
Self-induced	29/54 (53.7%)
Idiopathic	13/54 (24.1%)
Iatrogenic	9/54 (16.6%)
Coughing attack	3/54 (5.5%)
**Treatment**	
Medical	29/54 (53.7%)
Psychotherapy	14/54 (25.9%)
Behavioral	14/54 (25.9%)
Surgery	6/54 (11.1%)
Needle aspiration	2/54 (3.7%)
Sialendoscopy	2/54 (3.7%)
None	3/54 (5.5%)
**Resolution after Diagnosis (Days)**	4.5 ± 7.8
**Relapse**	
Yes	23/54 (42.6%)
No	28/54 (51.8%)
Unspecified	3/54 (5.5%)
**Complications**	
Subcutaneous emphysema	13/54 (24.1%)
Pneumomediastinum	3/54 (5.5%)
Abscess	1/54 (1.8%)
Parotitis	8/54 (14.8%)

*n*, number;%, percentage; M, media; SD, standard deviation.
